# An innovative surgical approach for solid pseudopapillary neoplasm with duodenal invasion in a pediatric patient: a case report

**DOI:** 10.1186/s40792-024-02047-w

**Published:** 2024-10-24

**Authors:** Yukihiro Tsuzuki, Kiyotetsu Ooshiro, Yukihiro Tatekawa, Rin Tamashiro, Takeshi Yagi, Takeshi Higa

**Affiliations:** 1 Department of Pediatric Surgery, Okinawa Nanbu Medical Center & Children’s Medical Center, 118-1, Arakawa, Shimajiri, Haebaru, Okinawa 901-1193 Japan; 2 Department of Pediatric Hematology/Oncology, Okinawa Nanbu Medical Center & Children’s Medical Center, 118-1, Arakawa, Shimajiri, Haebaru, Okinawa 901-1193 Japan

**Keywords:** Solid pseudopapillary neoplasm (SPN), Duodenal invasion, Enucleation, Pediatric cancer, Surgical techniques

## Abstract

**Background:**

Pediatric pancreatic tumors, especially with duodenal invasion, are exceptionally rare and a strategy for their treatment has not been established. A pancreaticoduodenectomy is often the desired treatment, but may be over-invasive for solid pseudopapillary neoplasm (SPN). This study reports an innovative surgical approach for SPN with duodenal invasion using pancreatic enucleation and endoscopically guided partial duodenectomy.

**Case presentation:**

An 11-year-old girl complained of malaise and presented with severe anemia; imaging revealed a tumor of undetermined origin, involving the pancreatic head and descending duodenum. Intraoperative findings showed tumor adherence to the pancreatic head and endoscopy revealed invasion of the duodenum. The tumor was enucleated from the pancreatic head, and partial duodenectomy was performed under endoscopically guided direct visualization. Pathology confirmed SPN with duodenal invasion, and no residual tumor. Although a Grade B pancreatic fistula occurred postoperatively, it was managed conservatively. At the 15-month follow-up, no signs of tumor recurrence, duodenal stenosis, or pancreatic dysfunction were evident.

**Conclusions:**

Given the good prognosis of SPN, we believe that enucleation from the pancreatic head combined with an endoscopically guided partial duodenectomy could be a useful and less invasive alternative to pancreaticoduodenectomy for cases with duodenal invasion.

## Background

Malignant pediatric pancreatic tumors are rare (0.018/100 000 in children aged 0–19 years) [1], and solid pseudopapillary neoplasms (SPNs) account for 17–57% of cases [2]. In 33% of patients the neoplasm is localized to the pancreatic head, but invasion of the surrounding area is rare, and duodenal invasion is uncommon [3]. SPN with duodenal invasion has no established treatment strategy. Although a pancreaticoduodenectomy (PD) is the most commonly used technique, we believe it may be over-invasive, especially for children. We report a case of SPN with duodenal invasion, treated with pancreatic enucleation and endoscopically guided partial duodenectomy.

## Case presentation

**Fig. 1:**
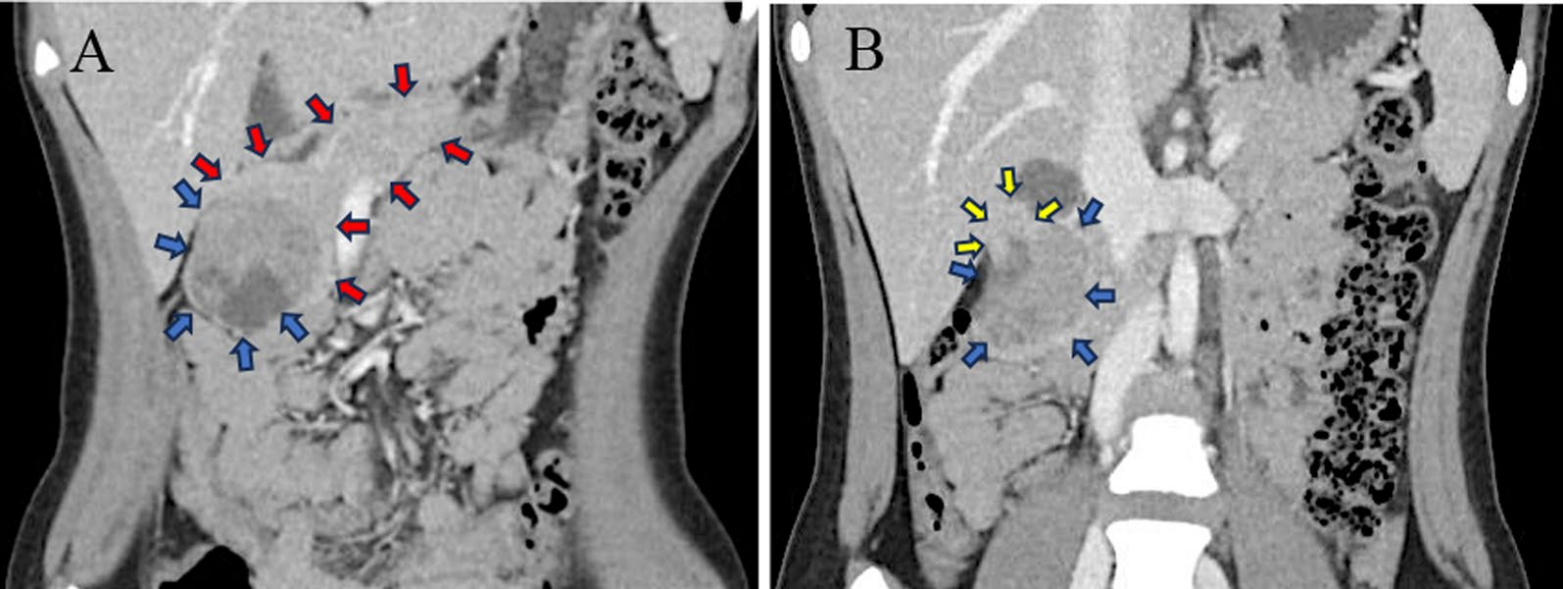
Images of preoperative MRI. Tumor (blue arrow) and pancreas (red arrow) (**A**). Tumor (blue arrow) and duodenum (yellow arrow) (**B**)

An 11-year-old girl with no significant past medical history experienced malaise 1 month before admission. Blood tests revealed iron deficiency anemia with Hb 4.6 g/dl, MCV 73.1 fL, Fe 6 μg/dL, ferritin < 5.0 ng/ml, reticulocyte 2.76%, TIBC 466 μg/dL. No elevated tumor markers or coagulation abnormalities were observed. Bone marrow aspiration showed no abnormalities. Magnetic resonance imaging (MRI) revealed a well-defined 47 × 50 mm tumor bordering the descending duodenum and pancreatic head (Fig. 1A and B). MR cholangiopancreatography showed no dilatation of the bile or pancreatic ducts, and the distance between the tumor and main pancreatic duct was 6 mm. Computed tomography (CT) revealed no enlarged regional lymph nodes or distant metastases. The images suggested a duodenal gastrointestinal stromal tumor (GIST), but differentiation from duodenal sarcoma, lymphoma, and pancreatic SPN was difficult.

**Fig. 2:**
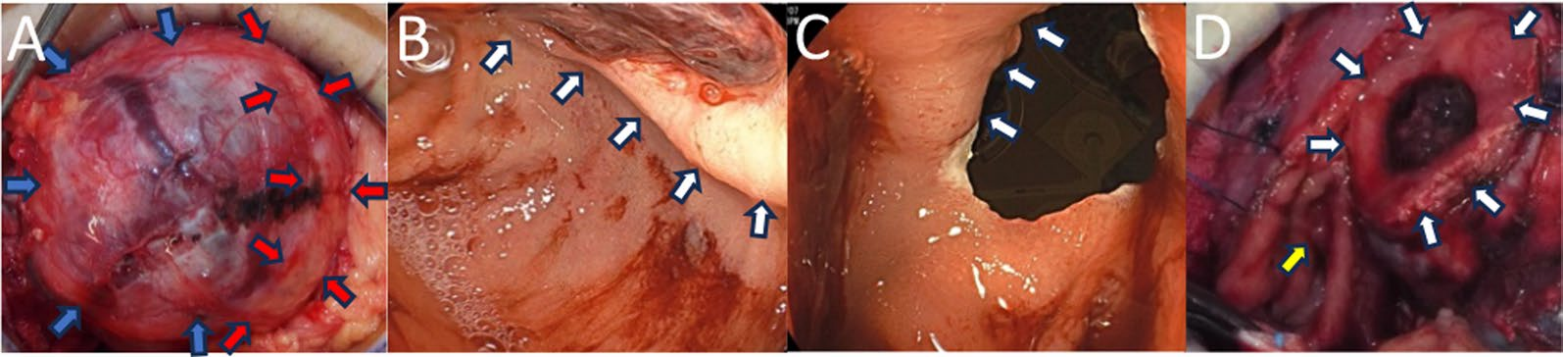
Intraoperative findings (**A**, **D**: surgical field, **B**, **C**: endoscope). The tumor (blue arrow) is adhered to the pancreatic head (red arrow) (**A**). Ulcer and marginal wall (white arrow) in the 2nd portion of the duodenum (**B**). Endoscopically guided resection of the tumor from the duodenum along the marginal wall (white arrow) and preservation of the papilla of Vater (yellow arrow) (**C**, **D**)

On laparotomy, the tumor was connected to the descending duodenum and pancreatic head (Fig. 2A). To prevent damage to the main pancreatic duct, we performed a combination of sharp and blunt dissection along the tumor capsule to minimize damage to the pancreatic parenchyma, and enucleated the tumor from the pancreas. Gastrointestinal endoscopy revealed an ulcer with marginal swelling in the descending duodenum (Fig. 2B). The tumor occupied 25% of the duodenal circumference. If the tumor were resected with minimal margins, the papilla of Vater could be preserved and the duodenal wall defect would not be large enough to cause stenosis. The tumor was resected under endoscopic guidance without damaging the papilla of Vater (Fig. 2C, D), the duodenal wall was sutured closed, and a closed drain was inserted into the resection site (duration: 162 min, blood loss: 90 ml, red blood cell transfusion volume: 280 ml).

Pathologically, round nuclei and eosinophilic stained tumor cells grew papillarily from the vascular core, with pseudorosettes, hemorrhage, necrosis, and cyst formation. The tumor was surrounded by a fibrous capsule; however, it had partially ruptured, exposing the tumor. Immunostaining was positive for β-catenin, CD56, and CD10, and negative for chromogranin A. The nuclear fission image was unclear (< 1/per high power field). Based on these findings, SPN was diagnosed. Although the capsule ruptured, the tumor was considered to have been completely resected based on the location of the rupture and intraoperative findings.

On postoperative day (POD)3, the ascitic amylase level was more than three times the serum value; therefore, pancreatic fistula was diagnosed. On POD8, the ascites amylase level dropped, and a fat-restricted diet was initiated. The patient was discharged on POD22. At 15 months postoperatively, there has been no apparent recurrence, abnormal glucose tolerance, or dyspepsia.

## Discussion

Although SPN is potentially malignant, 95% of patients are disease-free after surgical resection, and the mortality rate is less than 2%, indicating a good prognosis [3].

SPN with duodenal invasion is rare. Papaviramidis et al. reported that 497 patients with SPN had evidence of metastasis or invasion; 97 were positive with 27, 26, 17, and 27 in the liver, portal vein, spleen, and other organs including the duodenum, respectively [4]. Zhan et al. reported 91 cases of SPN, with 1 case (1.1%) of duodenal invasion [5]. Natsume et al. reported a case of SPN ruptured into the duodenum requiring PD [6]. Ours is the first report on resecting an SPN invading the duodenum using pancreatic enucleation and partial duodenectomy.

Although enucleation has been commonly performed for SPN of the pancreatic head recently, reports suggest that radical resection, including PD, should be performed in cases with local invasion or distant metastases [5]. But PD alters natural food passage leading to the mixing of oral nutrition with digestive enzymes, and the incidence of diabetes mellitus after surgery is 15–40%, pathologic exocrine pancreatic function varied from 22 to 55% due to the loss of healthy pancreatic parenchyma [7]. Enucleation is a simple procedure with short surgical duration, low blood loss, and good maintenance of pancreatic endocrine/exocrine function [8]. Only 2% of patients with SPNs have lymph node metastases [3]. Considering that SPNs are often low-grade and rarely have lymph node metastases, enucleation with a low risk of pancreatic endocrine/ exocrine dysfunction shows significant benefits, especially in children, in whom PD may be over-invasive. In this patient, enucleation from the pancreas allowed tumor resection with minimal pancreatic parenchyma loss, and using endoscopy, the duodenal side of the tumor could be resected precisely, avoiding extensive resection with PD and excessive reconstruction with gastrointestinal pathway alteration. We believe that a reduction surgery like this one should be considered when the tumor is expected to be completely resectable and damage to surrounding organs is anticipated to be acceptable, even for duodenal invasion of SPN. Specifically, the tumor should be located on the periphery of the pancreatic head, the size should allow for enucleation with a low risk of injuring the main pancreatic duct, neither enlarged lymph nodes nor invaded major blood vessels should be evident, and when the duodenal diameter after tumor resection and suturing should not be expected to cause stenosis. Particular attention should be given to the possibility of tumor recurrence after reduction surgery. The median time to recurrence is reported to be 4 years [3], so in this case, follow-up is planned for over 5 years using a combination of abdominal ultrasound and MRI.

We considered endoscopic ultrasound-guided fine needle aspiration (EUS-FNA) biopsy to confirm the diagnosis, but neoadjuvant chemotherapy has limited efficacy in the treatment of the suspected pediatric GIST [9], and the lesion would likely need to be resected to control the bleeding; therefore, primary surgery was selected. In a systematic review of 2744 patients with SPN, preoperative percutaneous or EUS-FNA biopsies were performed in 253 patients, and 64.8% were diagnostic of SPN [3]. EUS-FNA may be diagnostic, but its accuracy varies, and it is only recommended for unresectable cases [10]. In a US National Cancer Database study, biopsies were performed in 28.7% of pediatric patients with SPN [11]. Although preoperative biopsy is uncommon in children with suspected SPN, in this patient, tumor resection after pathological diagnosis may have avoided tumor capsule failure and prevented pancreatic fistula by identifying it as a pancreatic tumor, and allowing more careful dissection of the pancreatic side.

## Conclusions

We present an innovative surgical approach involving enucleation and endoscopically guided partial duodenectomy for SPN with duodenal invasion. In addition, we emphasize the potential role of preoperative biopsy in optimizing surgical planning and minimizing postoperative complications.

Consent was provided by the patient and her legal guardian.

## Data Availability

The datasets used and/or analyzed during the current study are available from the corresponding author on reasonable request.

## Abbreviations

SPN: Solid pseudopapillary neoplasm
GIST: Gastrointestinal stromal tumor
EUS-FNA: Endoscopic ultrasound-guided fine needle aspiration
PD: Pancreaticoduodenectomy
POD: Postoperative day
MRI: Magnetic resonance imaging
CT: Computed tomography
